# Current Evidence, Diagnostic Challenges, and Contemporary Management of Lymphocytic Colitis: A Case Report and Literature Review

**DOI:** 10.7759/cureus.116177

**Published:** 2026-09-13

**Authors:** Oziegbe Egbo, Ojevwe H Egbo, Faith I Omoregie, Favour A Onyeka

**Affiliations:** 1 Internal Medicine, Benson Idahosa University, Benin City, NGA; 2 Anatomical Pathology, Benson Idahosa University, Benin City, NGA; 3 Internal Medicine, Ripple Medical Centre, Benin City, NGA

**Keywords:** budesonide, chronic watery diarrhea, colonoscopy, histopathology, lymphocytic colitis, microscopic colitis

## Abstract

Lymphocytic colitis (LC) is a subtype of microscopic colitis characterized by chronic, typically non-bloody, watery diarrhea and increased intraepithelial lymphocytes despite a macroscopically normal or near-normal colon. This clinicopathological discordance creates a major diagnostic challenge because clinically important symptoms may occur without visible mucosal abnormalities, leading to missed diagnoses when colonic biopsies are not obtained. Although recognition of LC has increased, important uncertainties remain regarding its epidemiology, pathogenesis, medication associations, natural history, and optimal management, particularly in recurrent or treatment-dependent disease. This case report of a 39-year-old man and narrative review synthesizes contemporary evidence on LC, emphasizing epidemiology, pathogenesis, clinical presentation, diagnostic approach, histopathological criteria, treatment, and evidence gaps. Recent evidence supports budesonide as the preferred first-line therapy for symptomatic disease, while emerging real-world data suggest potential roles for advanced therapies in selected budesonide-refractory or budesonide-dependent patients. However, these data largely come from mixed microscopic colitis populations. Epidemiological evidence remains concentrated in North America and Europe, and data from Africa, including Nigeria, are sparse. Improved clinical recognition, standardized biopsy practices, consistent histopathological assessment, LC-specific therapeutic research, and geographically diverse epidemiological studies are needed to reduce diagnostic delay and improve long-term management.

## Introduction

Chronic watery diarrhea is a common clinical presentation with a broad differential diagnosis that includes celiac disease, inflammatory bowel disease, chronic infection, medication-related disorders, functional gastrointestinal disorders, and other gastrointestinal and systemic conditions [1]. Diagnostic evaluation can be particularly challenging when colonoscopy demonstrates no obvious mucosal abnormality. Microscopic colitis is an important example because diagnosis depends on histological examination of colonic biopsies despite a frequently normal or near-normal endoscopic appearance [2].

Microscopic colitis comprises two principal histological phenotypes: lymphocytic colitis (LC) and collagenous colitis (CC) [2]. LC is characterized primarily by increased intraepithelial lymphocytes (IELs) within the surface epithelium, with variable epithelial injury and lamina propria inflammation, without the substantially thickened subepithelial collagen band characteristic of CC [3,4]. Clinically, LC most commonly presents with chronic, watery, non-bloody diarrhea, while colonoscopy is often macroscopically unremarkable [3,4].

The apparently normal endoscopic appearance of LC is clinically important because it can lead to failure to obtain biopsies and consequently missed diagnosis. Patients may undergo repeated investigations or receive treatment for presumed functional gastrointestinal disorders despite having a histologically demonstrable inflammatory disorder [5,6]. Because LC is treatable, appropriate clinical suspicion and tissue diagnosis may shorten diagnostic delay and facilitate effective symptom control [7].

Despite increasing recognition of microscopic colitis, several aspects of LC remain incompletely understood. These include the mechanisms underlying disease development, the contribution of genetic and environmental factors, the significance of medication associations, the relationship between histological and clinical activity, and the optimal management of recurrent, treatment-dependent, or refractory disease [8-12]. These uncertainties are particularly relevant in regions where population-based epidemiological data and diagnostic pathology services remain limited.

This case report and narrative review critically examines contemporary evidence on LC, emphasizing evidence specific to LC versus that extrapolated from the broader microscopic colitis literature. It focuses on epidemiology, pathogenesis, clinical presentation, diagnostic challenges, histopathological criteria, current management, and priorities for future research. An illustrative case of LC is presented to contextualize the reviewed evidence and highlight the practical diagnostic and therapeutic challenges encountered in clinical practice, particularly in resource-limited settings.

## Case presentation

A 39-year-old man presented with a nine-month history of recurrent, watery, non-bloody, non-mucoid diarrhea, occurring at a frequency of three to five episodes per day, with an estimated volume of approximately 200 mL per episode. He described associated abdominal cramps predominantly localized to the epigastric region, radiating to the back. Stool passage was characteristically associated with weakness. Stools were bulky but not pale or greasy. There was no associated vomiting, nausea, or fever. He noted initial weight loss, from a pre-illness weight of 86 kg to a nadir of 77 kg, with partial spontaneous regain to 82 kg at presentation. The patient described temporary and partial relief of diarrhea with self-administered loperamide, though symptoms consistently recurred. He disclosed prior alcohol consumption, estimated at six units per week (predominantly small-volume gin), but had ceased alcohol use approximately nine months prior to presentation. He denied tobacco or illicit substance use. Past medical history was unremarkable for hypertension, diabetes mellitus, autoimmune disease, or thyroid disorder. He had a prior admission for evaluation of diarrhea. No formal diagnosis was established during those admissions. Drug history on presentation included rabeprazole, which was prescribed two weeks earlier following an upper gastrointestinal endoscopy for recurrent reflux symptoms, and intermittent self-administered loperamide. He was not on non-steroidal anti-inflammatory drugs (NSAIDs), antidepressants, or other medications commonly associated with microscopic colitis.

Physical examination

On examination, the patient was calm, alert, and oriented. He was afebrile (temperature 36.9°C), not pale, anicteric, acyanosed, or dehydrated, with no peripheral lymphadenopathy or pedal edema. Vital signs at initial presentation were as follows: blood pressure 120/80 mmHg, pulse rate 86 beats per minute (regular, normal volume), respiratory rate 21 breaths per minute, weight 82 kg. Chest examination revealed vesicular breath sounds bilaterally. Cardiovascular examination was essentially normal. Abdominal examination revealed a full abdomen moving with respiration, with tenderness in the right hypochondrial region and the left iliac fossa. Liver span was estimated at 16 cm. No splenomegaly was elicited. Digital rectal examination revealed satisfactory perianal hygiene, no masses, no anal tags, and no abnormalities of the prostate. Central nervous system examination was unremarkable.

Investigations

Initial laboratory investigations revealed normal hematological indices, with a hemoglobin concentration of 15 g/dL and packed cell volume of 47%. Fasting blood glucose was 81 mg/dL. Serum electrolytes were within normal limits except for a mildly reduced bicarbonate level of 20 mmol/L. Serum urea and creatinine were normal (15 mg/dL and 0.8 mg/dL, respectively). Urinalysis showed trace proteinuria.

Liver function tests showed mild elevations in alanine aminotransferase (88.6 U/L), aspartate aminotransferase (51.8 U/L), and gamma-glutamyl transferase (72.3 U/L), with reduced serum albumin (20.6 g/L) and normal total bilirubin (1.09 mg/dL). Hepatitis B surface antigen, anti-HCV antibody, and HIV serology were non-reactive on repeated testing. Stool microscopy and culture did not identify any enteric pathogens.

Abdominal ultrasound demonstrated hepatomegaly with a liver span of 17.8 cm and raised hepatic echotexture, consistent with hepatic steatosis; no evidence of portal hypertension or ascites was identified. Viral hepatitis was excluded serologically.

Upper gastrointestinal endoscopy demonstrated Los Angeles Grade C esophagitis and antral gastritis. Gastric biopsy histology confirmed severe chronic active non-atrophic Helicobacter pylori-associated pangastritis with no evidence of malignancy.

The patient had previously undergone colonoscopy at another facility for evaluation of chronic diarrhea; however, colonic biopsies were not obtained. Following the presentation to our facility, a repeat colonoscopy was performed, with examination from the anus to the cecum. The macroscopic appearance was entirely normal throughout the colon (Figure 1). Biopsies were obtained from multiple sites, including the ileocecal valve, cecum, ascending colon, transverse colon, sigmoid colon, and rectum. We collected three biopsy specimens from each site and submitted them for histopathological examination.

**Figure 1:**
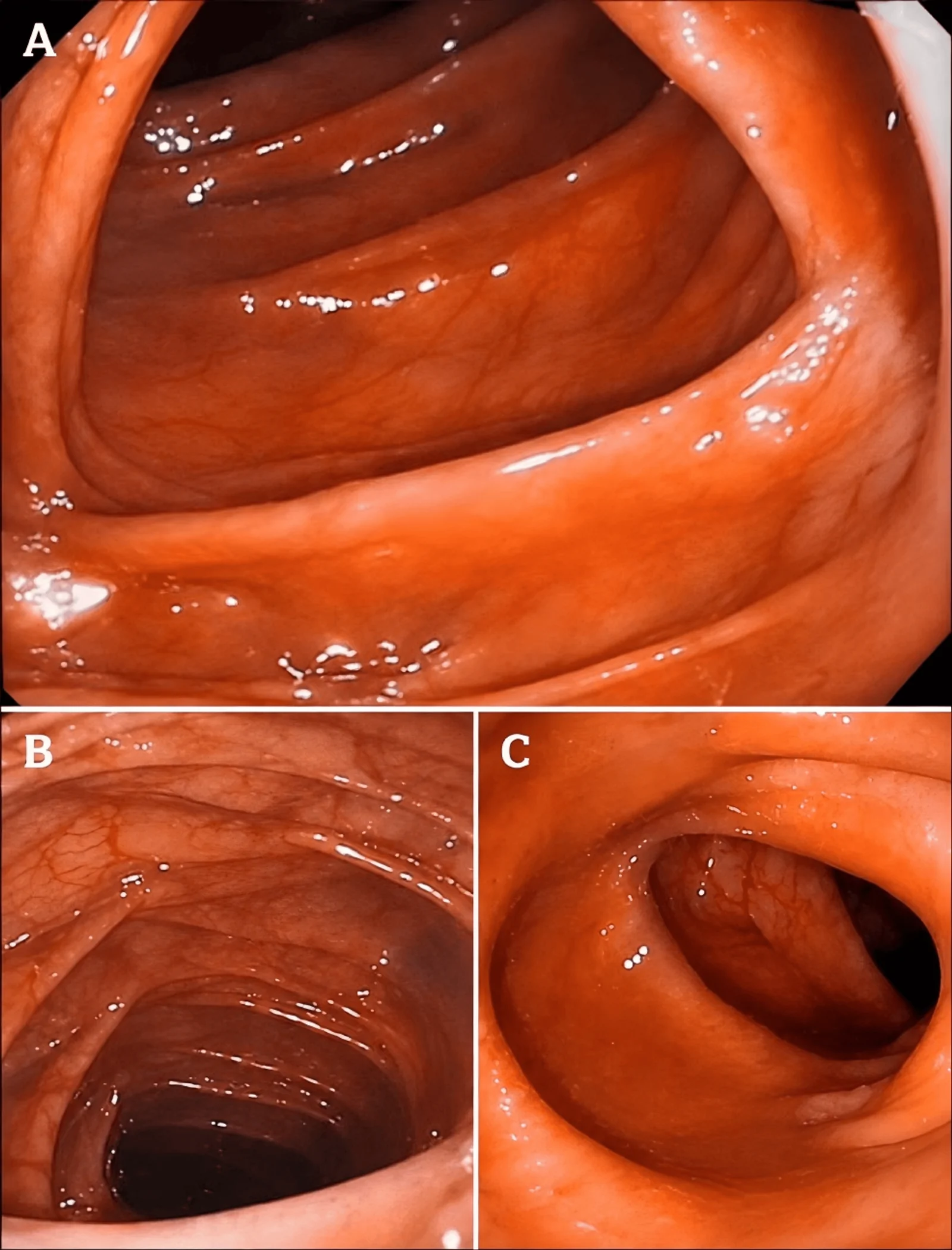
Colonoscopy images (A, B, and C) showing macroscopically normal colonic mucosa from multiple sites, including the transverse colon, sigmoid colon, and rectum

Literature search approach

We conducted a narrative literature search using PubMed/MEDLINE, Embase, and Google Scholar to identify literature on the epidemiology, pathogenesis, clinical presentation, diagnosis, histopathology, and treatment of LC. Search terms included combinations of “lymphocytic colitis”, “microscopic colitis”, “chronic watery diarrhea”, “epidemiology”, “pathogenesis”, “risk factors”, “diagnosis”, “histopathology”, “colonic biopsy”, “budesonide”, “relapse”, and “refractory disease”. We prioritized clinical guidelines, systematic reviews and meta-analyses, randomized controlled trials, population-based studies, and recent observational studies. We also screened reference lists of relevant reviews and guidelines to identify additional pertinent studies. We updated the literature search through August 2026. We placed particular emphasis on evidence specific to LC; where LC-specific evidence was limited, we considered relevant evidence from mixed microscopic colitis populations and explicitly noted this.

Epidemiology and global burden

LC predominantly affects middle-aged and older adults, with incidence increasing with age and a female predominance [13,14]. A systematic review and meta-analysis of 25 epidemiological studies estimated a pooled LC incidence of 4.85 per 100,000 person-years (95% CI, 3.45-6.25), with a female-to-male incidence rate ratio of 1.92 (95% CI, 1.53-2.31) and a median age at diagnosis of approximately 62 years [13]. A more recent systematic review and meta-analysis of 42 population-based studies involving more than 75 million individuals estimated a pooled LC incidence of 4.49 per 100,000 person-years (95% CI, 3.73-5.41) and a prevalence of 12.50 per 100,000 persons (95% CI, 5.36-29.16) [14].

Reported incidence and prevalence vary substantially between populations. In Olmsted County, Minnesota, for example, LC incidence was 15.8 per 100,000 person-years between 2011 and 2019, while prevalence reached 146.1 per 100,000 persons in 2019 [15]. Differences in case ascertainment, access to colonoscopy and histopathology, study design, population characteristics, and temporal changes in diagnostic awareness may contribute to this variation [14-16]. The markedly higher estimates from some regional cohorts should therefore not automatically be interpreted as evidence of true biological differences in disease frequency.

Epidemiological evidence from Africa and other low- and middle-income regions remains considerably less developed than that from North America and Europe [16,17]. A recent Nigerian colonoscopy-based study identified histologically confirmed LC in 21 of 122 individuals undergoing colonoscopy [17]. Although this finding demonstrates that LC occurs in the Nigerian population, its single-center, colonoscopy-based design does not permit estimation of disease incidence or population prevalence. The finding should therefore be interpreted as a colonoscopy-based series rather than a population prevalence estimate. More population-based studies from Africa and other underrepresented regions are needed to determine whether apparent differences in disease frequency reflect genuine epidemiological variation, differences in environmental exposures, or disparities in diagnostic recognition and access to histopathological assessment [14,16,17].

Pathogenesis and risk factors

The pathogenesis of LC is not fully established but is considered multifactorial. Current evidence supports interactions among mucosal immune dysregulation, epithelial barrier abnormalities, luminal and environmental exposures, genetic susceptibility, and potentially medication exposure [16,18,19]. Importantly, much of the mechanistic literature concerns microscopic colitis as a whole, so it remains unclear how specific these pathways are to LC.

Immune activation appears central to the disease phenotype. The characteristic accumulation of IELs, predominantly T-cells, provides histological evidence of cell-mediated mucosal inflammation [2,16,19]. Proposed mechanisms include altered epithelial-immune signaling and increased mucosal responsiveness to luminal stimuli, but researchers have not definitively identified the initiating antigens or environmental triggers [18,19].

Alterations in the intestinal microbiome have also been described in microscopic colitis [20,21]. However, many microbiome studies include both LC and CC, making it difficult to determine whether particular microbial alterations are specific to LC or represent shared features of microscopic colitis. Additionally, the direction of the relationship remains uncertain because dysbiosis may contribute to disease development or may occur secondary to intestinal inflammation or its treatment [20,21].

Genetic susceptibility may also influence disease development, although the genetic architecture of LC remains incompletely defined. Earlier studies reported associations between LC and selected HLA variants, including HLA-DR3-DQ2, while other investigations identified differences in HLA class I phenotypes [22,23]. More recent genome-wide and HLA-focused data involving patients with microscopic colitis did not demonstrate the same strength of HLA association in LC as in CC [24]. These findings suggest that genetic susceptibility may differ between microscopic colitis phenotypes, although the available datasets remain relatively small [24].

The association between LC and autoimmune disease further supports altered immune susceptibility. Celiac disease is particularly well established, with a systematic review and meta-analysis demonstrating an association between celiac disease and microscopic colitis, with an estimated prevalence of celiac disease of approximately 6.3% among patients with LC [25]. Other autoimmune conditions, including autoimmune thyroid disease and type 1 diabetes mellitus, have also been associated with microscopic colitis [26,27]. These associations support clinical screening for relevant coexisting autoimmune disease rather than implying a single autoimmune mechanism for LC.

Smoking represents another environmental risk factor. A case-control study found an increased risk of microscopic colitis among current smokers, including an adjusted odds ratio of 3.81 for LC [28]. Subsequent prospective cohort data similarly demonstrated increased risk among current and former smokers, although the association was stronger for CC than LC [29]. The consistency of the association supports smoking as a relevant modifiable exposure, although observational designs cannot establish that smoking is directly causal.

Medication exposure has frequently been investigated in relation to microscopic colitis. Proton pump inhibitors, non-steroidal anti-inflammatory drugs, and selective serotonin reuptake inhibitors are among the medication classes most consistently associated with the condition [2,30]. European guidelines acknowledge increased odds of microscopic colitis with exposure to these drugs, although associations are less consistent when LC is considered separately [2]. Differences in study design, exposure definitions, comparator groups, concomitant medications, confounding by indication, and assessment of symptom response after withdrawal limit conclusions regarding individual drug causality [2,30].

Overall, LC is best regarded as a multifactorial inflammatory disorder in which immune susceptibility interacts with environmental, luminal, genetic, and potentially medication-related factors [16,18,19]. The principal unresolved issue is not whether these factors are associated with LC, but how they interact to produce the characteristic epithelial lymphocytosis and clinical phenotype. LC-specific mechanistic studies, particularly those integrating molecular, microbiome, and immune data, may help identify biologically meaningful subgroups and future therapeutic targets.

Clinical presentation and patient burden

LC most commonly presents with chronic, watery, non-bloody diarrhea, which may be intermittent or continuous. In a Swedish cohort of 199 patients with histologically confirmed LC, diarrhea occurred in 96% of patients, abdominal pain in 47%, weight loss in 41%, nocturnal diarrhea in 39%, fatigue in 21%, and fecal incontinence in 9% [31]. Other symptoms, including urgency, bloating, and increased intestinal gas, may also occur [2,31].

Disease severity varies substantially. Some patients experience intermittent symptoms, whereas others develop frequent and persistent diarrhea that interferes with sleep, work, social activities, and daily functioning [2,31]. Urgency and fecal incontinence can be particularly disruptive and may contribute to social restriction and psychological distress. Persistent diarrhea may also result in repeated healthcare visits and investigations. Because the clinical phenotype overlaps with functional gastrointestinal disorders, symptoms alone cannot reliably distinguish LC from other causes of chronic watery diarrhea.

Diagnostic approach

Clinical Suspicion and Initial Evaluation

LC should be considered in adults with chronic or recurrent watery, non-bloody diarrhea, particularly when accompanied by fecal urgency, nocturnal stools, abdominal discomfort, fecal incontinence, fatigue, or weight loss [31,32]. Clinical assessment should establish the duration and pattern of diarrhea. It should include review of stool characteristics, nocturnal symptoms, weight change, medication exposure, dietary factors, previous gastrointestinal disease, and relevant family history [1].

Medication history is particularly important because several drug classes have been associated with microscopic colitis. However, these associations do not necessarily establish causality. When a suspected drug has a plausible chronological relationship with the onset of diarrhea, withdrawal may be considered, with the decision individualized according to the clinical context and the need to avoid unnecessary interruption of beneficial therapy [2].

The differential diagnosis remains broad and includes functional diarrhea, diarrhea-predominant irritable bowel syndrome, celiac disease, chronic infection, inflammatory bowel disease, bile acid diarrhea, pancreatic insufficiency, endocrine disorders, and medication-related diarrhea [1]. The clinical overlap with functional gastrointestinal disorders is particularly relevant because some patients with LC may initially be labeled as having irritable bowel syndrome with diarrhea (IBS-D) when appropriate colonoscopic biopsy is not performed [2,6].

Initial Laboratory and Stool Evaluation

Laboratory and stool investigations primarily aim to identify alternative or coexisting causes of chronic diarrhea rather than establish LC itself [1]. Routine laboratory tests are often normal or show nonspecific abnormalities, and no validated biomarker can reliably establish or exclude microscopic colitis [2]. Initial testing should therefore narrow the differential diagnosis while avoiding unnecessary investigations that delay definitive tissue assessment.

Stool investigations should be selected based on the clinical context and may include microbiological testing for bacterial, parasitic, or other infectious causes [1]. Testing for celiac disease should be considered because of its recognized association with microscopic colitis, and appropriate serological testing should be recommended in patients undergoing evaluation for suspected disease [2,25].

Bile acid diarrhea is another important differential diagnosis and may coexist with microscopic colitis. Bile acid diarrhea should be considered particularly when diarrhea persists despite apparently adequate treatment of LC [2]. Other investigations for malabsorption, pancreatic insufficiency, endocrine disease, or medication-related diarrhea should be guided by the clinical presentation rather than performing them indiscriminately [1].

Fecal calprotectin may be elevated in some patients with microscopic colitis but has limited diagnostic utility and cannot reliably exclude the disease [2]. A normal fecal calprotectin concentration should therefore not by itself eliminate LC from consideration when the clinical presentation remains compatible [2].

Colonoscopy and Adequate Biopsy Sampling

The principal diagnostic value of colonoscopy in suspected LC is the opportunity to obtain colonic biopsies for histopathological assessment. A macroscopically normal colon does not exclude LC [1,2]. Thus, when microscopic colitis is suspected, the endoscopist should view colonoscopy primarily as a tissue-acquisition procedure rather than a search for characteristic macroscopic lesions.

Therefore, biopsies should be obtained even when the mucosa appears normal. Current guidance recommends sampling from both the right and left colon rather than relying on rectal or limited distal biopsies because histological abnormalities may vary between colonic segments [1,2]. Multiple biopsies from different colonic sites improve diagnostic yield when abnormalities are patchy or unevenly expressed [2,33,34].

The anatomical sites of biopsy specimens should be documented, and right- and left-sided biopsies should be submitted in separately labeled containers where feasible to facilitate pathological interpretation, particularly in borderline cases [2]. Inadequate or limited sampling may reduce diagnostic yield [2].

Histopathological criteria

Diagnostic Features of Lymphocytic Colitis

Histopathological examination remains the diagnostic cornerstone of LC. The defining abnormality is an increased number of IELs, conventionally ≥20 IELs per 100 surface epithelial cells, accompanied by variable lamina propria inflammation and epithelial injury [3,4,8]. Unlike CC, LC lacks a substantially thickened subepithelial collagen band [3,4,8].

The intraepithelial lymphocytic population in LC is predominantly T-cell derived, particularly CD3+/CD8+ T-cells [35]. Assessment should focus specifically on the surface epithelium and distinguish IELs from lymphocytes within the lamina propria or crypt epithelium [3,4].

Surface Epithelial and Lamina Propria Changes

Surface epithelial injury may accompany increased IELs and can include epithelial flattening, mucin depletion, surface irregularity, degeneration, and focal epithelial detachment [3,4]. The severity and distribution of these abnormalities may vary between biopsy specimens and colonic segments [4]. The lamina propria commonly contains an increased chronic inflammatory infiltrate predominantly composed of lymphocytes and plasma cells, while crypt architecture is generally preserved [3,4,8].

Distinction from Collagenous Colitis

LC and CC share clinical and histological features but differ in their dominant abnormalities. LC is characterized by prominent surface epithelial lymphocytosis without a substantially thickened subepithelial collagen layer. In contrast, CC is characterized by a thickened, irregular subepithelial collagen layer, usually thicker than 10 μm and often with an irregular or ragged appearance, along with variable epithelial injury and lamina propria inflammation [3,4,8,36].

Some histological overlap exists between LC and CC, with increased IELs occurring in CC and occasional histological transition between the two phenotypes [4]. These findings support the concept of microscopic colitis as a related group of disorders while also highlighting why histological criteria should be applied consistently when classifying individual biopsies.

H&E and Immunohistochemistry

Routine hematoxylin and eosin staining is generally sufficient for diagnosing LC when the characteristic increase in surface IELs can be demonstrated [3,4]. Immunohistochemistry may be useful in selected diagnostically difficult cases, with CD3 staining highlighting the predominantly T-cell intraepithelial population. However, routine immunohistochemistry is unnecessary when characteristic findings are readily identified on H&E sections [35].

Borderline and Incomplete Microscopic Colitis

Sampling, tissue quality, biopsy orientation, IEL-counting methodology, and pathological experience may influence histological interpretation, particularly in borderline or incomplete cases [2-4]. Although the IEL threshold provides an important diagnostic benchmark, interpretation should integrate IEL distribution and morphology, epithelial injury, lamina propria inflammation, collagen-band thickness, and relevant clinical information [3,4].

Increased IELs are not specific to LC and may be encountered in conditions such as celiac disease, resolving infection, medication-associated mucosal injury, and inflammatory bowel disease [3,4,25]. This lack of specificity is particularly relevant in cases with borderline IEL counts, in which the diagnosis should be based on the complete histological pattern and clinical context [3,4].

Some patients demonstrate histological abnormalities suggestive of microscopic colitis without fulfilling the conventional diagnostic criteria for LC or CC. These cases may be categorized as incomplete microscopic colitis. Incomplete LC is characterized by an increased IEL count (>10 but <20 IELs per 100 surface epithelial cells), a normal subepithelial collagen band, and mild lamina propria inflammation [2]. Because these findings may represent an incomplete form of the characteristic pattern, clinicopathological correlation remains important, particularly when counts are close to the diagnostic threshold [2-4].

Table 1 summarizes the histopathological features used to distinguish LC, CC, and incomplete LC.

**Table 1:** Histopathological features used to distinguish LC, CC, and incomplete LC LC: lymphocytic colitis, CC: collagenous colitis, IELs: intraepithelial lymphocytes

Feature	LC	CC	Incomplete LC
IELs	≥20 per 100 surface epithelial cells	May be increased	>10 but <20 per 100 surface epithelial cells
Subepithelial collagen band	Not substantially thickened	Thickened or irregular; usually >10 μm, often with an irregular or ragged appearance	Normal
Lamina propria	Usually increased chronic inflammation	Usually increased chronic inflammation	Mild inflammation
Surface epithelial injury	Variable	Variable	Usually mild
Crypt architecture	Generally preserved	Generally preserved	Generally preserved

Diagnostic challenges

Normal Endoscopic Appearance and Failure to Biopsy

The macroscopically normal or near-normal colon in LC can make the condition difficult to recognize. Clinically significant watery diarrhea may occur without visible mucosal abnormalities, and failure to biopsy an apparently normal colon can lead to missed or delayed diagnosis [1,2,6].

Inadequate Sampling and Sampling Error

Obtaining biopsies does not eliminate the possibility of a false-negative or equivocal diagnosis if the number, distribution, or orientation of samples is inadequate [2,34]. Histological abnormalities may vary between colonic segments, while poor tissue orientation may make assessing the surface epithelium and IEL density difficult [2,34]. Sampling error is therefore an important consideration when histological findings are negative or borderline despite a clinical presentation strongly suggestive of LC. Consistent sampling of the right and left colon, appropriate specimen orientation, and clear documentation of biopsy sites may improve diagnostic reliability [2,34].

Histological Overlap and Interpretation

Increased IELs are not pathognomonic for LC and may occur in celiac disease, resolving infection, medication-associated mucosal injury, inflammatory bowel disease, and other inflammatory conditions [3,4,25]. The diagnostic challenge is therefore not simply to identify an elevated IEL count but to determine whether the distribution and degree of epithelial lymphocytosis, together with epithelial and lamina propria changes, form a coherent pattern of LC.

Variability in biopsy orientation, tissue quality, counting methodology, and pathological experience may further contribute to diagnostic uncertainty [2-4]. Standardized criteria and communication between endoscopists and pathologists may improve diagnostic consistency.

Underdiagnosis and Delayed Diagnosis

Nonspecific symptoms, normal endoscopic findings, incomplete biopsy practices, and challenges in interpreting histology can delay diagnosis [6]. Patients may be labeled as having IBS-D or functional diarrhea when microscopic colitis has not been adequately excluded because colonoscopic biopsies were not obtained [6].

This challenge may be particularly relevant in resource-limited settings, including sub-Saharan Africa, where limited awareness and access to specialized endoscopic and histopathological expertise may restrict recognition of microscopic colitis [17]. In such settings, underdiagnosis may reflect health-system constraints as much as disease rarity.

Importantly, histological disease activity does not necessarily parallel clinical disease activity. A multicenter study found that histological activity in microscopic colitis was not significantly associated with clinical disease activity or one-year disease course [9]. Accordingly, clinical symptoms remain central to routine assessment, and repeat colonoscopy or biopsy is generally unnecessary solely to document treatment response [2,9].

Contemporary management

Removal of Potentially Implicated Medications and Lifestyle Measures

Management should begin by assessing potentially reversible contributors to diarrhea. Review and withdraw medications associated with microscopic colitis, including proton pump inhibitors, non-steroidal anti-inflammatory drugs, and selective serotonin reuptake inhibitors, when clinically appropriate, particularly when a convincing temporal relationship exists between exposure and symptom onset [2].

Medication withdrawal should nevertheless be individualized, as epidemiological associations between these drugs and microscopic colitis do not establish causality in every patient [2]. Smoking cessation should also be encouraged because smoking is associated with microscopic colitis [2].

Dietary and Supportive Measures

Coexisting causes of persistent diarrhea, particularly celiac disease and bile acid diarrhea, should be considered and treated when identified [2,25]. Symptomatic therapy may be appropriate for patients with mild disease [2,37]. Antidiarrheal agents such as loperamide may reduce stool frequency in selected patients but are primarily symptomatic and should generally be used as adjunctive rather than disease-modifying therapy [2,37].

First-Line Pharmacological Therapy

Budesonide is the best-supported first-line pharmacological treatment for symptomatic LC [2,37]. The American Gastroenterological Association recommends budesonide to induce clinical remission in symptomatic microscopic colitis and favors it over mesalamine for induction [37].

In a randomized, double-blind, placebo-controlled trial specifically involving LC, budesonide 9 mg daily for six weeks produced clinical remission in 86% of treated patients compared with 48% of patients receiving placebo [7]. Histological remission was also significantly more frequent with budesonide [7].

The broader evidence base supports budesonide as the preferred induction treatment across microscopic colitis [38], with a commonly recommended regimen of 9 mg daily for approximately six to eight weeks. Individualize treatment duration based on clinical response and disease course [2,37].

Management of Recurrent or Refractory Disease

Patients who do not respond adequately to initial therapy should first undergo reassessment of the diagnosis, adherence, medication exposure, and possible coexisting causes of diarrhea [2,37]. Bile acid diarrhea, celiac disease, infection, medication-related diarrhea, and functional gastrointestinal disorders should be reconsidered where clinically appropriate [2].

Bile acid sequestrants such as cholestyramine may be considered when bile acid diarrhea is demonstrated or strongly suspected [2,37]. Other agents, including bismuth subsalicylate and mesalamine, have been studied, but the evidence supporting their use is less robust than that for budesonide [37].

Immunomodulators such as azathioprine and 6-mercaptopurine have been used in selected patients with severe, chronic, or budesonide-dependent disease, although limited evidence and potential toxicity restrict their routine use [2,37]. Their role is best considered in specialist care after confirming the diagnosis and excluding coexisting causes of persistent diarrhea.

Maintenance Therapy and Relapse

Relapse after discontinuation of budesonide is common [11,38]. In the randomized LC trial, approximately 44% of patients experienced clinical relapse during follow-up after budesonide withdrawal [7]. Broader microscopic colitis studies similarly demonstrate a substantial risk of relapse following discontinuation and support maintenance treatment in patients with recurrent disease [11,38].

Patients with infrequent relapses may be retreated with budesonide when symptoms recur [2,37]. Those with frequent relapses or budesonide dependence may require longer-term maintenance therapy using the lowest effective dose [2,11,37].

The available maintenance evidence is derived predominantly from microscopic colitis populations and includes substantial representation of CC [11]. Although this limits the certainty with which the findings can be extrapolated to LC, the overall evidence supports maintenance budesonide in appropriately selected patients with recurrent disease [2,11].

Long-Term Follow-Up

Long-term management should balance sustained symptom control against unnecessary corticosteroid exposure [2,11]. A 2024 systematic review and meta-analysis reported high rates of clinical remission during budesonide maintenance and a substantial relapse rate after treatment discontinuation [11].

Patients requiring prolonged therapy should have periodic reassessment of the ongoing need for treatment and potential corticosteroid-related adverse effects [2]. Bone health should be considered in patients receiving prolonged budesonide therapy, and calcium and vitamin D supplementation may be considered when clinically appropriate, particularly in those with additional osteoporosis risk factors [2].

Routine repeat colonoscopy and biopsy are not generally required solely to assess treatment response [2,9]. Clinical response should therefore remain the principal outcome used for routine follow-up.

Emerging and Advanced Therapies

Biologic therapy may have a role in highly selected patients with severe, refractory microscopic colitis who remain symptomatic despite optimized conventional treatment [39]. Reported treatments include infliximab, adalimumab, and vedolizumab, but available evidence largely consists of case reports, case series, and small observational studies [39].

Most available studies combine LC and CC, limiting the ability to determine the efficacy of biologic therapy specifically in LC [39]. Consequently, biologics should not be considered routine therapy for uncomplicated LC and should generally be reserved for carefully selected refractory or treatment-dependent cases managed in specialist settings [2,39].

Evidence for small-molecule therapies is evolving. A 2026 multinational retrospective study from the ECCO CONFER network evaluated 229 advanced-therapy treatment cycles in 142 patients with microscopic colitis after budesonide failure or intolerance; 47.2% of the cohort had LC [40]. JAK inhibitors were associated with the highest short-term clinical response and remission rates (95.2% and 81.0%, respectively) and lower discontinuation than other advanced therapies [40]. These findings are important because they provide substantially more real-world evidence than earlier reports, but they should not be interpreted as establishing JAK inhibitors as standard LC therapy. The study was retrospective, used advanced therapies off-label, and reported outcomes for microscopic colitis as a combined LC and CC population despite including both LC and CC [40]. Prospective LC-specific comparative trials are therefore needed before advanced therapies can be incorporated into routine treatment algorithms.

Current evidence gaps and future directions

Uncertainty Regarding Pathogenesis

Although current evidence supports a multifactorial immune-mediated process, the mechanisms responsible for epithelial lymphocytosis, mucosal immune activation, medication susceptibility, and differences between microscopic colitis phenotypes remain incompletely understood [4,19]. Future mechanistic studies should distinguish LC-specific pathways from mechanisms shared with CC and should integrate immune, epithelial, genetic, and microbiome data.

Need for Standardized Diagnostic Criteria

The threshold of 20 IELs per 100 surface epithelial cells provides a useful diagnostic benchmark, but borderline cases remain challenging [3,4,8]. Differences in biopsy orientation, sampling location, counting methodology, and pathological interpretation can influence classification [2-4,8]. Greater standardization of biopsy protocols, histological assessment, reporting terminology, and criteria for incomplete microscopic colitis could improve diagnostic consistency and facilitate cross-study comparisons [2-4,8].

Limited Evidence from Low- and Middle-Income Countries

Available population-based epidemiological evidence remains concentrated in North America and Europe, with limited data from other regions [14]. Nigerian data demonstrate that LC occurs locally but remain insufficient to establish its population-level burden [17].

Future studies in Nigeria and other low- and middle-income countries should examine disease frequency, clinical phenotype, diagnostic practices, pathology capacity, treatment accessibility, affordability, and long-term clinical outcomes. Such research could determine whether the apparent scarcity of LC reflects genuinely lower prevalence or under-recognition and underdiagnosis [17].

Long-Term Treatment and Maintenance Strategies

Despite budesonide's effectiveness for induction, uncertainty remains about the optimal duration of maintenance therapy, the lowest effective maintenance dose, relapse prevention, and the long-term safety of repeated or prolonged treatment [7,11,38]. LC-specific trials are particularly needed because much of the available maintenance and advanced-therapy evidence is derived from broader microscopic colitis populations, including patients with CC [2,11,40].

Biomarkers and Non-invasive Diagnostic Approaches

Non-invasive inflammatory markers, including fecal calprotectin, lack sufficient diagnostic accuracy to establish or exclude microscopic colitis and therefore cannot replace histological assessment [2]. Future research should investigate biomarkers that can identify active LC, predict treatment response, monitor disease activity, and distinguish LC from other causes of chronic watery diarrhea. Reliable non-invasive approaches could potentially reduce the need for repeated invasive investigation and improve diagnostic efficiency, but any proposed biomarker will require validation in well-characterized LC cohorts.

Taken together, the major research priorities are LC-specific epidemiological studies, standardized histopathological assessment, validation of non-invasive biomarkers, and prospective clinical trials addressing induction, maintenance, relapse prevention, and refractory disease (Table 2) [2,4,11,39,40].

**Table 2:** Contemporary therapeutic options for LC Evidence beyond budesonide remains limited and is often extrapolated from mixed microscopic colitis populations. CC: collagenous colitis, LC: lymphocytic colitis, TNF: tumor necrosis factor, JAK: Janus kinase

Therapeutic option	Typical role	Evidence strength	Key limitation
Budesonide	First-line induction; maintenance for recurrent disease	Strongest evidence	Relapse is common after withdrawal; long-term use requires individualized risk assessment
Loperamide/symptomatic therapy	Adjunctive symptom control in selected mild disease	Limited/supportive	Not disease-modifying
Bile acid sequestrants	When bile acid diarrhea is demonstrated or strongly suspected	Selected patients	Treats a coexisting mechanism rather than LC itself
Mesalamine/bismuth	Alternative or selected cases	Limited	Inferior or less consistently supported compared with budesonide
Azathioprine/6-mercaptopurine	Selected severe, chronic, budesonide-dependent disease	Very limited	Potential toxicity and specialist use
Biologics (anti-TNF, vedolizumab, ustekinumab)	Refractory or treatment-dependent disease	Low; mainly observational	Most evidence combines LC and CC
JAK inhibitors	Emerging option for highly refractory disease	Emerging 2026 real-world evidence	Off-label; retrospective data and mixed microscopic colitis populations

## Discussion

Overall, this case provides a practical clinical illustration of the evidence presented in this review. Our patient's nine-month history of recurrent watery, non-bloody diarrhea, abdominal cramps, weakness, and associated weight loss was compatible with the clinical spectrum of LC described in the literature [31,32]. However, his age and sex are less typical of the demographic profile in which microscopic colitis is most frequently recognized. This reinforces the importance of considering LC based on clinical presentation rather than excluding the diagnosis because of demographic characteristics.

Despite the characteristic clinical presentation, the initial endoscopic evaluation did not establish a diagnosis. The diagnosis was established only after repeat colonoscopy and systematic biopsies from multiple colonic segments. This sequence illustrates how LC may remain undiagnosed when histological sampling is omitted despite an apparently normal colonoscopic examination.

The histological findings strongly supported the diagnosis. The presence of 24 IELs per 100 surface epithelial cells, together with preserved crypt architecture and absence of a substantially thickened subepithelial collagen band, was consistent with the histopathological criteria for LC.

A key practical feature of this case was the difficulty of obtaining budesonide. This illustrates how internationally supported recommendations may be difficult to implement in resource-limited settings. The case therefore extends the discussion beyond treatment efficacy to treatment accessibility. Where appropriate diagnostic biopsy and effective therapy are not readily available, patients with LC may experience prolonged symptoms, repeated healthcare encounters, and delayed diagnosis.

There are limitations to this case. Molecular stool testing was not performed, and bile acid diarrhea and pancreatic exocrine insufficiency were not formally evaluated. Long-term follow-up and post-treatment histological assessment were also limited. These factors mean that the case cannot establish that every potential alternative cause of diarrhea was definitively excluded. Nevertheless, the combination of the characteristic clinical presentation, extensive colonic biopsy sampling, diagnostic histology, and marked response to budesonide provides a coherent clinical picture of LC.

## Conclusions

LC is an important and treatable cause of chronic watery diarrhea that may remain unrecognized because substantial gastrointestinal symptoms can occur despite a normal or near-normal colonoscopic appearance. Appropriate evaluation therefore requires clinical consideration of LC and adequate colonic biopsy sampling, even when no macroscopic abnormality is identified. Histopathological assessment remains central to diagnosis, while interpretation should account for sampling adequacy, segmental variation, and overlap with other causes of epithelial lymphocytosis.

Budesonide remains the preferred first-line treatment for symptomatic disease, whereas recurrent, treatment-dependent, and refractory LC continue to present important management challenges. The evidence base is also limited by the frequent inclusion of LC and CC within the same studies, particularly for maintenance and advanced therapies. Improved clinical recognition, standardized biopsy and histopathological practices, geographically diverse epidemiological research, and LC-specific therapeutic studies are needed to reduce diagnostic delay and strengthen the evidence base for long-term management.
